# Simulation of Health and Economic Benefits of Extended Observation of Resolved Anaphylaxis

**DOI:** 10.1001/jamanetworkopen.2019.13951

**Published:** 2019-10-23

**Authors:** Marcus Shaker, Dana Wallace, David B. K. Golden, John Oppenheimer, Matthew Greenhawt

**Affiliations:** 1 Section of Allergy and Immunology, Dartmouth-Hitchcock Medical Center, Lebanon, New Hampshire; 2 Dartmouth Geisel School of Medicine, Hanover, New Hampshire; 3 Nova Southeastern Allopathic Medical School, Fort Lauderdale, Florida; 4 Division of Allergy-Clinical Immunology, Johns Hopkins University, Baltimore, Maryland; 5 Department of Internal Medicine, UMDMJ-Rutgers New Jersey Medical School, Newark; 6 Children’s Hospital Colorado, University of Colorado School of Medicine, Section of Allergy and Immunology, Food Challenge and Research Unit, Aurora

## Abstract

**Importance:**

Biphasic anaphylaxis may occur in up to 20% of patients with anaphylaxis; however, the optimal observation time of patients with resolved anaphylaxis is unknown.

**Objective:**

To characterize the cost-effectiveness of short vs prolonged medical observation times after resolved anaphylaxis.

**Design, Setting, and Participants:**

An economic evaluation was performed of computer-simulated adult patients observed in outpatient allergy clinics and emergency departments, with rates of biphasic anaphylaxis derived from a 2019 meta-analysis.

**Exposures:**

Computer-simulated patients (10 000 per strategy) were randomized to undergo 1 hour of medical observation (associated with 95% negative predictive value of biphasic anaphylaxis) or 6 or more hours of observation (associated with a 97.3% negative predictive value of biphasic anaphylaxis).

**Main Outcomes and Measures:**

Cost-effectiveness of 6- to 24-hour medical observation of resolved anaphylaxis evaluated at willingness-to-pay thresholds of $10 000 per case of biphasic anaphylaxis observed and $10 million per death prevented, assuming that observation is associated with a 10- to 1000-fold reduction in the risk of death due to biphasic anaphylaxis.

**Results:**

Biphasic anaphylaxis occurred after hospital discharge in 365 patients observed for 1 hour and in 213 patients undergoing prolonged observation. From a health care sector perspective, with medical observation costs of $286.92 per hour, the incremental cost of extended medical observation of resolved anaphylaxis (1 hour vs 6 hours) was $62 374 per case of biphasic anaphylaxis identified ($68 411 from the societal perspective). In Monte Carlo simulations, with hourly costs ranging from $100 to $500 and extended observation ranging from 6 to 24 hours (health care sector perspective), the mean (SD) costs were $295.36 ($81.22) for 1 hour of observation vs $3540.42 ($1626.67) for extended observation. The incremental cost-effectiveness ratio was $213 439 per biphasic anaphylaxis observed ($230 202 from the societal perspective). A 6-hour observation could be cost-effective if the risk of biphasic anaphylaxis after 1-hour observation of resolved anaphylaxis was 17% or if hourly observation costs were less than $46 in the base case. Cost-effectiveness could also be achieved (willingness-to-pay of $10 million per death prevented, health care sector perspective) when a baseline fatality rate of 0.33% per biphasic anaphylactic event was assumed, with a no greater than 24% relative risk of fatality associated with 6-hour observation.

**Conclusions and Relevance:**

This study indicates that prolonged medical observation (6-24 hours) for resolved anaphylaxis may not be cost-effective for patients at low risk for biphasic anaphylaxis; however, in particular clinical circumstances of low observation costs, high postdischarge risk of biphasic anaphylaxis, or large incremental fatality risk reduction associated with extended observation, longer medical observation can be justified.

## Key Points

**Question:**

What is the cost-effectiveness of 1 hour vs 6 to 24 hours of medical observation for biphasic anaphylaxis?

**Findings:**

In this economic evaluation, routine use of prolonged medical observation costs $62 374 to $230 202 per case of biphasic anaphylaxis observed, depending on the hourly costs of observation and duration of stay, with costs of prolonged observation approaching or exceeding $10 million per death prevented.

**Meaning:**

Routine use of 6- to 24-hour medical observation for unselected patients with resolved anaphylaxis is a low-value medical practice unless the risk of biphasic anaphylaxis is significantly pronounced.

## Teaser

This economic evaluation uses computer-simulated patients to examine the cost-effectiveness of short (1 hour) vs prolonged (≥6 hours) medical observation for biphasic anaphylaxis after an episode of resolved anaphylaxis.

## Introduction

Anaphylaxis is an acute event that represents a life-threatening emergency.^1^ Although estimates vary, the lifetime prevalence of anaphylaxis ranges between 1.6% to 5.1%, with an incidence of 42 cases per 100 000 person-years.^1,2,3,4^ Leading causes of anaphylaxis include medications, foods, and stinging insects, although many cases are idiopathic.^1,2,5,6,7,8,9^ Fatal anaphylaxis is rare, with an overall estimated prevalence of 0.47 to 0.69 cases per million persons.^10,11,12^ Although anaphylaxis-associated hospitalizations have increased, case-fatality rates have remained stable at 0.25% to 0.33% of hospitalizations or emergency department (ED) visits for anaphylaxis.^13^

Biphasic anaphylaxis is a potential sequela of resolved anaphylaxis, by definition occurring after anaphylaxis has been treated and completely resolved for at least 1 hour.^14,15^ Biphasic anaphylaxis can be life-threatening and has been reported to occur up to 78 hours after the initial episode of anaphylaxis.^15^ It is important to distinguish biphasic anaphylaxis from a protracted (incompletely responsive) initial anaphylactic episode, as well as to differentiate repeated anaphylactic episodes from subsequent reexposure to an unidentified trigger.^16,17^ Biphasic anaphylaxis may occur in less than 1% to 20% of individuals with anaphylaxis, and its occurrence is difficult to predict with any certainty.^14,18,19^ Specific treatments for biphasic anaphylaxis have not been well studied, but at present, standard practice focuses on management with rapid administration of intramuscular epinephrine—management identical to the treatment of index anaphylaxis.^15,20^ Although antihistamines and glucocorticoids are frequently used in an attempt to prevent biphasic anaphylaxis, clear evidence supporting a benefit associated with this practice is scant.^16,20,21,22,23^ In the community setting, epinephrine autoinjectors in the United States are now administered as a twin-pack (2 devices) only, secondary to the risk of a poorly responsive primary reaction or the risk of a biphasic reaction, and individuals are advised to carry both units on their person at all times.^24^

Because the percentage of individuals who may be at risk for biphasic anaphylaxis could vary widely, there is no clear consensus regarding how long someone presenting to an ED or outpatient allergy clinic should be observed after they are treated, and a 4- to 6-hour observation time is commonly used, although there is no evidence to suggest that this time is appropriate or necessary.^24^ The question regarding the length of observation is particularly timely, given that recent evidence suggests that immediate presentation to an ED or activation of emergency medical services (EMS) in the community setting after a food allergic reaction treated with epinephrine (vs epinephrine administration followed by watchful waiting for improvement, activating EMS only for a lack of response to treatment) was a very low-value service. The cost of seeking attention immediately regardless of response is in excess of $1 billion over the time horizon to prevent 1 fatality. Furthermore, it was only marginally cost-effective when simulated over greatly exaggerated fatality risk rates (>100 fold) attributable to not immediately seeking emergency care.^25^ Although many standard community anaphylaxis management plans may stress prompt activation of EMS and observation in an ED after epinephrine administration, irrespective of patient response and degree of stability, it is unclear whether these actions deliver a high-value medical service or they are overly cautious and cost-ineffective. After treatment of the index anaphylaxis event, there is a wide variation in the perceived mandatory or even needed time of observation to ensure complete symptom resolution and to monitor for a potential biphasic response. Therefore, there is a need to understand both the health benefits and the economic burden of an extended observation time compared with a shortened observation time, to determine the most appropriate advisory recommendations for postanaphylaxis management.^26,27^ As such, we undertook a cost-effectiveness analysis to characterize value-based practice of brief vs more prolonged observation times of adult patients with resolved anaphylaxis.

## Methods

### Decision Model

A cost-effectiveness analysis of extended observation was performed using a simple decision tree that incorporated hourly costs of observation (2019 US dollars) and risks for biphasic anaphylaxis associated with a 1-hour symptom-free interval compared with a 6-hour or longer symptom-free interval.^27^ TreeAge Pro software version 2018 (TreeAge Software) was used to construct a decision tree (eFigure 1 in the Supplement) comparing the strategy of a 1-hour observation following complete resolution of anaphylaxis without recurrence in that observation time with the more prolonged strategy (≥6 hours). Cohort analyses using base-case values and Monte Carlo simulations (MCS) incorporating inputs (10 000 computer-simulated patients per strategy)^28,29,30,31^ with triangular modal distributions across plausible ranges were performed (Table 1). Triangular distributions were represented by the mode of each variable bounded by the minimum and maximum assumptions fitted to a triangle, with maximum apical unit distribution at the mode and probability minimized at extreme values, and were validated by alternative beta distributions for probability of anaphylaxis and fatality and gamma distributions for costs and observation time. A 78-hour time horizon was used to evaluate the cost of observation per case of biphasic anaphylaxis identified. Given the short time horizon, the model was not adjusted for the discount rate of future dollars or life-years, and age-adjusted all-cause mortality was not included.

**Table 1.** Model Inputs

Variable	Cohort Input (Simulation Range)^a^	Source
Cost per hour of observation, $^b^	286.92 (100-500)	Blumenthal et al, 2018,^28^ and Bureau of Labor Statistics^29^
Indirect costs per hour, $^b^	27.77 (0-50)	Bureau of Labor Statistics^30^
Extended observation, h	6 (6-24)	Kim et al, 2019^31^
Short observation, h	1	
Biphasic anaphylaxis negative predictive value at 1-h observation, %	95 (95-99)	
Biphasic anaphylaxis negative predictive value at ≥6-h observation, %	97.3 (97.3-99)	
Biphasic anaphylaxis case fatality, %	0.33 (0.25-0.33)	Ma et al, 2014^13^
Fatality risk reduction	10-fold	Modeling assumption (sensitivity range 10- to 1000-fold)

^a^ The cohort input also served as the mode for the triangular distribution used in simulation.

^b^ Costs are shown in 2019 US dollars.

This simulation did not involve human participants and, per the Colorado Multiple Institutional Review Board, did not require review or an exemption from review because it used previously published inputs and hypothetical cohorts of patients with resolved anaphylaxis. No identified or deidentified patient information was included in data sets used in the cohort analysis or simulations. This study follows the Consolidated Health Economic Evaluation Reporting Standards (CHEERS) reporting guideline.^32^

### Biphasic Anaphylaxis Risks and Observation Times

Model inputs were derived from a recent meta-analysis^27^ that was performed to evaluate the risks for biphasic anaphylaxis in association with observation time after resolution of an initial anaphylactic reaction. Twelve studies including 2890 adult patients with anaphylaxis and 143 patients with biphasic reactions were included in the meta-analysis,^27^ with the negative predictive value (NPV) for postdischarge biphasic anaphylaxis calculated for 1-hour and more prolonged observation periods. Details of the meta-analysis are described elsewhere.^27^ Briefly, of 175 studies identified, 12 were included in the analysis (dates of publication, 1986-2018; mean patient age range, 30.2-55.8 years), inclusive of anaphylaxis caused by drug, food, insect bite, iodinated contrast mediated, and idiopathic causes. Rates of biphasic anaphylaxis ranged from 0.4% to 20%, with the mean interval between the initial anaphylaxis resolution and the secondary reaction ranging from 1.75 to 33 hours. The pooled NPV of 1-hour observation was 95%, with the NPV of 6-hour or longer observation reported to be 97.3%.^27^ The cohort rate of postdischarge biphasic anaphylaxis was 5.0% (1-hour observation) and 2.7% (extended observation of ≥6 hours). The MCS biphasic risk was derived from 5.0% and 2.7% modal sample distributions (range, 1%-5% for 1 hour and 1%-2.7% for extended observation). In the cohort analysis, 1-hour observation was compared with 6-hour observation, whereas in the MCS, 1-hour observation was compared with 6-hour triangular modal population distributions (range, 6-24 hours).

### Costs

Model costs were based on prior studies^28,33^ of time-driven activity-based costing, which is a bottom-up costing approach to evaluate combined resource costs within health care as a factor of utilization. Yu and colleagues^33^ recently described a time-driven activity-based costing approach to value assessment in pediatric appendicitis. In their study,^33^ preintervention ED time for care delivery was 269 minutes with a cost of $296.21 ($0.91 per minute). Blumenthal et al^28^ recently reported on costs for care delivery within an allergy clinic in the context of penicillin allergy evaluation using a time-driven activity-based costing model. Model costs were based on the analysis by Blumenthal et al^28^ to include hourly costs of the examination room, allergist or immunologist, registered nurse, and medical assistant, with sensitivity costs incorporating ranges reported by Yu et al^33^ (eTable in the Supplement). The impact of job-related opportunity costs for patients recently discharged after treatment of anaphylaxis is uncertain and variable; therefore, the analysis was performed both from a societal perspective including job-related opportunity costs and from a health care sector perspective (HCP) with indirect costs excluded.^34^ The cohort hourly cost for observation was $286.92, and the MCS hourly observation cost was sampled from a modal distribution set at this value (range $100-$500).

### Outcomes

Costs were expressed in 2019 US dollars. Cost-effectiveness of medical observation was evaluated by cost per biphasic anaphylaxis medically observed. The incremental cost-effectiveness ratio was calculated by the difference in cost between 1-hour and prolonged observation divided by the difference in rates of observed biphasic anaphylaxis while under medical supervision. Given the additional complexities and uncertainties in downstream costs and risks of biphasic anaphylaxis in different settings, additional cost-utility analyses were not performed in this analysis; however, cost per fatality prevented was evaluated using a 10- to 1000-fold a priori risk-reduction calculation in the rate of anaphylaxis fatality, assuming a threshold of $10 million per death prevented, which is the federal estimate of the value of a statistical life.^35^ Using the Nationwide Emergency Department Inpatient Sample (1999-2009), the Nationwide Emergency Department Sample (2006-2009), and Multiple Cause of Death Data (1999-2009), Ma et al^13^ evaluated the case fatality rate among hospitalization and ED visits for patients presenting with anaphylaxis from the general population and reported case fatality rates between 0.25% and 0.33% among hospitalizations or ED presentations for anaphylaxis as the principal diagnosis. These rates translated to 63 to 99 deaths per year in the United States.^13^ Both inpatient and outpatient fatality rates were less than 1 per 1 million population. The cohort analyses incorporated a fatality risk of 0.33% per biphasic anaphylaxis occurring at home with a 10- to 1000-fold risk reduction for biphasic anaphylaxis identified under medical observation, with the MCS sampling modal (0.33%) distributions between 0.25% and 0.33% fatality risk.^13^

### Statistical Analysis

Descriptive statistics were used to evaluate means with SDs for outcome variables. Deterministic and probabilistic sensitivity analyses were performed on all inputs. Specific exploratory analyses were performed to explore the risk difference between 1-hour and more prolonged observation, which could make the practice of extended observation cost-effective, as well as the hourly medical observation cost that could create a value-based paradigm of care. Evaluations were performed using more extended willingness-to-pay (WTP) thresholds of $50 000 to $100 000 per biphasic anaphylaxis observed. Statistical analysis was performed using TreeAge Pro software.

## Results

### Cost per Biphasic Anaphylaxis Observed

#### Health Care Perspective

Biphasic anaphylaxis occurred after hospital discharge in 365 patients observed for 1 hour and 213 patients undergoing prolonged observation. In the cohort analysis with observation time limited to 6 hours and costs held at $286.92 per hour, the cost of extended medical observation beyond 1 hour after anaphylaxis resolution was $62 374 per additional biphasic anaphylaxis observed (Table 2). In the MCS, the mean (SD) cost for 1 hour of observation was $295.36 ($81.22), whereas that for extended observation (6-24 hours) was $3540.42 ($1626.67). After discharge, a mean (SD) of 3.7% (1.0%) of patients experienced a biphasic reaction after 1 hour of observation compared with 2.1% (0.4%) of patients in the 6- to 24-hour observation group. In the simulation, the incremental cost per biphasic anaphylaxis observed was $213 439 (Table 2).

**Table 2.** Cost-effectiveness Results, Health Care Sector Perspective

Strategy	Cost, $US^a^	Rate of Biphasic Anaphylaxis After Discharge, %	Incremental Cost Per Additional Biphasic Anaphylaxis Identified Under Medical Observation, $
Cohort analysis			
1-h observation	286.92	5.0	62 374
6-h observation	1721.52	2.7	
Monte Carlo simulation, mean (SD)			
1-h observation	295.36 (81.22)	3.7 (1.0)	213 439
≥6-h observation	3540.42 (1626.67)	2.1 (0.4)	

^a^ Costs are shown in 2019 US dollars.

#### Societal Perspective

The cost of observation was $314.69 for 1 hour and $1888.14 for 6 hours in the cohort analysis, with the resulting cost per biphasic anaphylaxis observed estimated at $68 411. In the simulation with indirect costs included, the incremental cost per biphasic anaphylaxis observed was $230 202. The mean (SD) cost of an extended 6 -to 24-hour observation time was $3875 ($1730) vs $322.67 ($82.91) for 1 hour of observation. Evaluation of alternate distribution simulations demonstrated that 6-hour observation cost $68 701 per biphasic anaphylaxis observed, with 24-hour observation costing $312 455 per biphasic anaphylaxis observed.

#### Sensitivity Analyses

**Figure 1.**
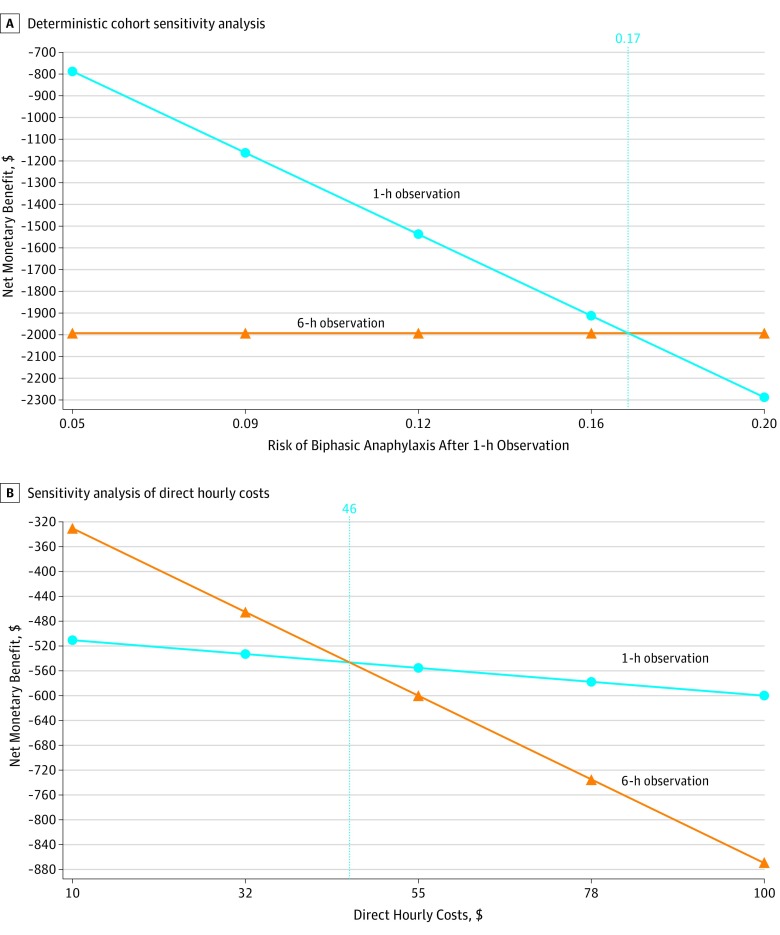
Sensitivity Analysis (Cost per Biphasic Anaphylaxis Episode Observed) A, Deterministic sensitivity analysis of risk for biphasic anaphylaxis (after complete resolution) is shown. If the risk for biphasic anaphylaxis exceeds 17% (dotted vertical line), then extended observation for 6 hours would cost less than $10 000 per case of biphasic anaphylaxis identified. B, Sensitivity analysis of direct hourly costs is shown. If the direct hourly cost of observation is less than $46 (dotted vertical line), 6-hour observation could be cost-effective (willingness-to-pay = $10 000 per observed biphasic anaphylaxis episode).

**Figure 2.**
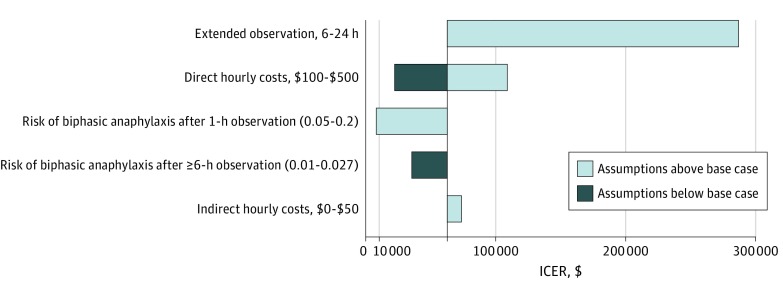
Tornado Diagram of Deterministic Sensitivity Analyses Incremental cost-effectiveness ratio (ICER) is shown in 2019 US dollars (cost per biphasic anaphylaxis episode observed). Tornado diagram shows deterministic sensitivity analyses, with light blue bars representing assumptions above and dark blue bars representing assumptions below the base case analysis.

**Figure 3.**
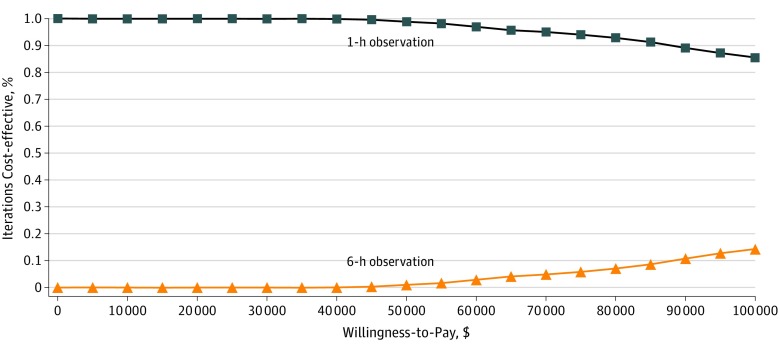
Cost-effectiveness Acceptability Curve (Cost per Biphasic Anaphylaxis Episode Observed) Probabilistic sensitivity analysis (1000 simulations) is shown. Across modal distributions, extended observation (≥6 hours) is not cost-effective compared with 1-hour observation after anaphylaxis resolution at a willingness-to-pay of $10 000 per biphasic anaphylaxis episode observed.

Deterministic cohort sensitivity analysis (HCP) indicated that 6-hour observation could be cost-effective (WTP = $10 000 per biphasic anaphylaxis observed) if the benefit of 1-hour asymptomatic medical observation was much poorer than reported (ie, the risk of a biphasic after discharge exceeded 5%) (Figure 1). If the risk of biphasic anaphylaxis after 1-hour postanaphylaxis resolution exceeded 17%, then a more extended strategy of 6 hours became cost-effective at a WTP of $10 000 per biphasic anaphylaxis observed. With 1-hour observation associated with a 95% NPV of biphasic anaphylaxis, extended observation of 6 hours did not become cost-effective unless the hourly cost of observation was $46 or less (indirect costs excluded) (Figure 1). From the HCP, valuation of a 4-hour extended observation period cost $37 424 per observed biphasic anaphylaxis ($41 047 from the societal perspective). Deterministic sensitivity analyses across additional variables did not demonstrate cost-effectiveness of extended observation (Figure 2). In probability sensitivity analyses (1000 simulations) across modal distributions (Figure 3), extended observation was not cost-effective in any simulation (WTP = $10 000 per additional biphasic anaphylaxis identified under medical observation).

### Cost per Fatality Prevented

#### Health Care Perspective

With a 10-fold reduction in anaphylaxis fatality associated with 6-hour observation in the cohort analysis, the cost compared with 1-hour observation was $9 190 851 per death prevented ($8 699 243 with 1000-fold fatality risk reduction). In the MCS, the cost per death prevented by the extended observation (6-24 hours) with a 10-fold fatality risk reduction was $31 061 955 ($29 056 105 at 1000-fold risk reduction).

#### Societal Perspective

With indirect costs included in the cohort analysis, the cost of 6- vs 1-hour medical observation of resolved anaphylaxis with a 10-fold fatality risk reduction was $10 080 402 per death prevented ($9 541 213 with 1000-fold fatality risk reduction). In the 6- to 24-hour simulation with indirect costs included, the incremental cost per death prevented was $33 386 290 for a 10-fold fatality risk and $31 704 472 for a 1000-fold fatality risk. Evaluation of alternate distribution simulations demonstrated 6-hour observation costs of $10 333 425 per death prevented, with 24-hour observation costing $44 337 332 per death prevented.

#### Sensitivity Analyses

Cohort sensitivity analysis from the HCP indicated that 6-hour observation could be cost-effective (WTP = $10 million per death prevented) if 6-hour observation was associated with no greater than a 24% relative risk of death from biphasic anaphylaxis, compared with 1-hour observation (≤9% relative risk of death from the societal perspective). See eFigure 2 in the Supplement for details.

## Discussion

Anaphylaxis can be a life-threatening event, and there is a risk of poor response to initial therapy requiring care escalation, as well as the risk of a biphasic reaction occurring hours after an initial event was successfully treated and resolved. For this reason, persons at risk for anaphylaxis are recommended to carry at least 2 units of self-injectable epinephrine at all times, are recommended to seek emergency care after epinephrine is used, and are commonly observed for several hours after an event.^24^ However, evidence substantiating the cost-effectiveness of these practices is limited. Indeed, this analysis indicates that extended observation (beyond 1 hour) of resolved anaphylaxis may not be cost-effective (assuming a WTP of $10 000 per biphasic anaphylaxis observed), unless the risk for biphasic anaphylaxis exceeds 17% after a 1-hour symptom-free interval (vs 2.7% after 6 hours), or hourly observation costs are less than $46 for up to 6 hours (with indirect costs excluded). Cost-effectiveness could also be achieved (WTP of $10 million per death prevented) from the HCP with a baseline fatality rate of 0.33% per biphasic anaphylactic event, assuming a 24% relative risk of fatality associated with more prolonged ED observation. Although a threshold of both cost and risk can be determined to justify commonly cited observation times, whether this degree of protection could be obtained with prolonged observation is questionable. In patients with resolved anaphylaxis without high risk factors for a biphasic reaction or anaphylaxis fatality, a 1-hour period of observation may be reasonable.

This is the first study, to our knowledge, to explore the health and economic outcomes regarding postanaphylaxis medical observation times for patients receiving treatment in an ED. Coupled with earlier work that questions the value of activating EMS for stable patients (even after having received epinephrine for a severe allergic reaction) who do not worsen under at-home observation, this does represent a potential opportunity to reevaluate anaphylaxis care, recognizing that the specter of costly services (both in terms of time and money spent) for such EMS activation and prolonged ED observation may serve to deter some patients from using epinephrine in cases of severe reactions.^25^ For low-risk patients with resolved anaphylaxis, ongoing medical observation could potentially represent an area of preference-sensitive care where shared decision-making could be of value. In instances where either patients or clinicians are less comfortable with at-home management or are less familiar and experienced with anaphylaxis or epinephrine use, or postdischarge access to epinephrine or medical care is questionable, extended observation may still be reasonable for lower risk patients. Likewise, extending the observation time for resolved anaphylaxis from 6 to 24 hours may represent a cost-ineffective strategy for many patients. However, in some circumstances and settings, prolonged observation may be clinically warranted and health and economic outcomes justified, and there could be consideration for preference-sensitive care in certain scenarios regarding shorter vs more prolonged observation time. Specific situations that may be identified in the future that could support more extended observations include patients with risk factors associated with a higher rate of biphasic anaphylaxis after discharge (ie, requiring multiple doses of epinephrine or receiving epinephrine after a considerable time delay, such as >30-60 minutes), a greater risk for fatality associated with biphasic anaphylaxis (ie, comorbidities such as cardiac or respiratory disease), or low costs of medical observation and job-related opportunity costs.^17,36,37^

### Limitations

Cost-effectiveness analyses are based on modeling and assumptions, many of which have variation and can be debated. This analysis, accordingly, has several limitations. First, there is uncertainty and variation in regional costs associated with observation and the clinical context of observation. Second, the inputs are largely based on a single meta-analysis, which lacked randomized clinical trials (this is a limitation of anaphylaxis research, in general), and, therefore, was based on observational data that could have multiple inherent biases, the cumulative result of which could affect the assumptions used for this model. To account for this, very broad plausible ranges were considered in the multiple sensitivity analyses we conducted. Third, model costs were based primarily on utilization of allergy clinic resources, and the costs of ED observation could be greater. Fourth, it is difficult to exclude more vulnerable subgroups who may benefit from extended observation or situations in which patient preference–sensitive care may factor into decisions for more prolonged observation; thus, the analysis did not incorporate such considerations. Fifth, because the time horizon of observation was short, Markov analysis was not performed to evaluate ongoing probabilistic risk for biphasic anaphylaxis beyond 78 hours (or medical observation beyond 24 hours). Sixth, data were not available on risk reduction from medical observation between 1 and 5 hours, and, as such, the current analysis did not extensively evaluate cost-effectiveness of medical observation within this time window. Seventh, the precise WTP threshold for biphasic anaphylaxis detection under observation is unknown. Cost-effective care is defined as $50 000 to $100 000 per quality-adjusted life-year,^38^ with the statistical value of a life estimated at $10 million.^35^ From a societal perspective for most patients, routine use of prolonged medical observation (6-24 hours) for resolved anaphylaxis costs $68 411 to $230 202 per case of biphasic anaphylaxis detected by observation. However, the cost of more extended observation ranges from $10 080 402 to $33 386 290 per death prevented (societal perspective). Within these limitations, evidence suggests that, for low-risk patients with completely resolved anaphylaxis, 1-hour observation may be a reasonable and high-value strategy provided that patients are educated regarding the risk of biphasic anaphylaxis and that they understand the thresholds for further care.

## Conclusions

The present analysis is consistent with prior evidence suggesting that routine prolonged medical observation of completely resolved episodes of anaphylaxis, to prevent a biphasic reaction, is not cost-effective.^25^ As the Joint Taskforce on Allergy Practice Parameters (representative of the American Academy of Allergy Asthma and Immunology and the American College of Allergy Asthma and Immunology) completes its work on a Grading of Recommendations, Assessment, Development, and Evaluation–based anaphylaxis practice parameter,^39^ these findings should support evidence-based guidance for postanaphylaxis observation times. Although all patients with anaphylaxis should be educated regarding risks for biphasic reactions and understand home management and thresholds for further care, those patients with risk factors that indicate a greater likelihood of biphasic anaphylaxis or higher fatality risk may benefit from more extended observation of up to 6 hours (and possibly longer in some circumstances). Conversely, it may be reasonable to discharge low-risk patients after a 1-hour observation period following resolved anaphylaxis.

## Notes

Supplement. **eFigure 1.** Decision Model **eFigure 2.** Sensitivity Analysis (Cost per Death Prevented) **eTable.** Time-Dependent Activity-Based Costing Estimates
