# Supplementary material for: Simulation of Health and Economic Benefits of Extended Observation of Resolved Anaphylaxis
Source: JAMA Netw Open. 2019 Oct 23;2(10):e1913951. doi: 10.1001/jamanetworkopen.2019.13951 (PMC6820064; doi:10.1001/jamanetworkopen.2019.13951)
Supplement: Supplement. — eFigure 1. Decision Model eFigure 2. Sensitivity Analysis (Cost per Death Prevented) eTable. Time-Dependent Activity-Based Costing Estimates [file jamanetwopen-2-e1913951-s001.pdf]

Supplementary Online Content

Shaker M, Wallace D, Golden DBK, Oppenheimer J, Greenhawt M. Simulation of health and economic benefits of extended observation of resolved anaphylaxis. *JAMA Netw Open*. 2019;2(10):e1913951. doi:10.1001/jamanetworkopen.2019.13951

eFigure 1. Decision Model
eFigure 2. Sensitivity Analysis (Cost per Death Prevented)
eTable. Time-Dependent Activity-Based Costing Estimates

This supplementary material has been provided by the authors to give readers additional information about their work.

© 2019 Shaker M et al. *JAMA Network Open.*

eFigure 1. Decision Model

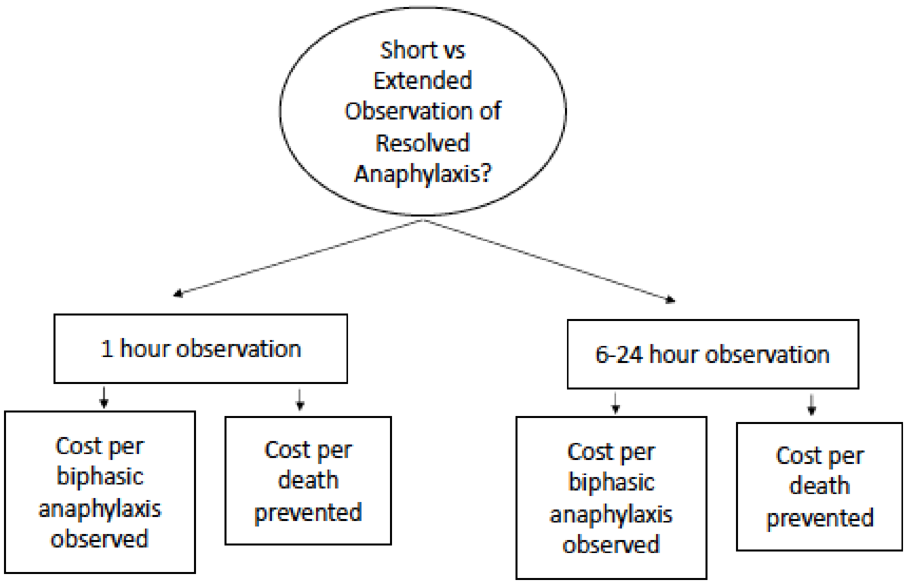

Legend: Decision Model of 1-hour vs 6 to 24-hour post-anaphylaxis resolution observation time.

© 2019 Shaker M et al. *JAMA Network Open.*

eFigure 2. Sensitivity Analysis (Cost per Death Prevented)

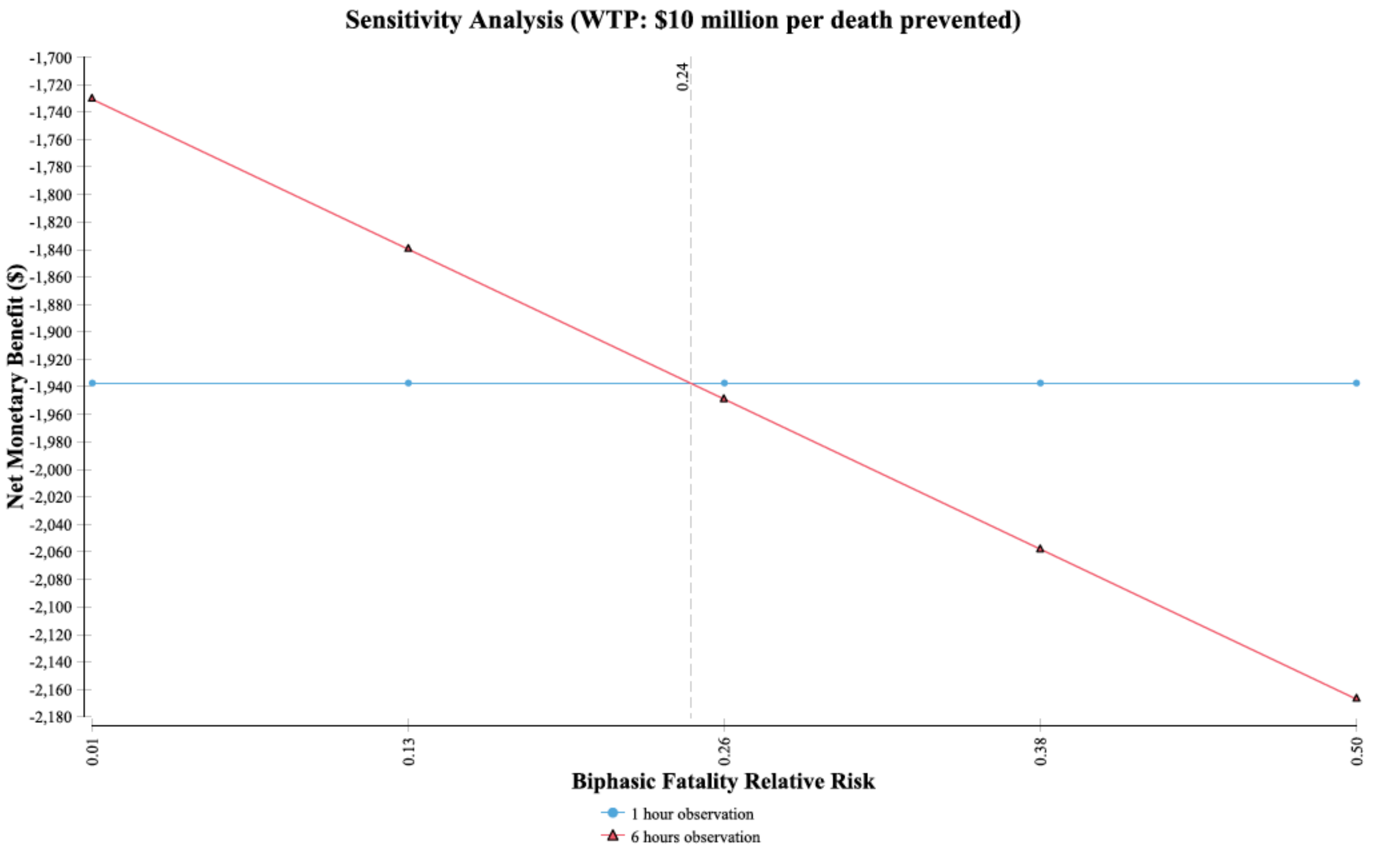

Legend: Deterministic sensitivity analysis of fatality relative risk for biphasic anaphylaxis (following complete resolution). If the relative risk for biphasic anaphylaxis is 24% or less (HCP perspective), then extended observation for 6 hours would cost less than $10 million dollars per death prevented ($\leq$ 9% from the societal perspective)

© 2019 Shaker M et al. *JAMA Network Open.*

eTable. Time-Dependent Activity-Based Costing Estimates

Resource	Cost per minute (2019 dollars)	Cost per hour (2019 dollars)
Exam Room	$0.03	$1.92
Allergist/Immunologist	$3.62	$217.20
Registered Nurse	$0.78	$46.80
Medical assistant	$0.35	$21.00
Total		$286.92

© 2019 Shaker M et al. *JAMA Network Open.*
